# Concurrent manifestation of clinical hypodontia and blindness: a case report

**DOI:** 10.15171/joddd.2017.010

**Published:** 2017-03-15

**Authors:** Jeremiah Robert Moshy, Karpal Singh Sohal, Mark Chindia

**Affiliations:** ^1^Senior Lecturer, Department of Oral and Maxillofacial Surgery, Muhimbili University of Health and Allied Sciences, Dar-es-Salaam, Tanzania; ^2^Postgraduate Student, Department of Oral and Maxillofacial Surgery, Department of Dental Services, Muhimbili National Hospital, Dar-es-Salaam, Tanzania; ^3^Professor, Department of Oral and Maxillofacial Surgery, Oral Pathology and Oral Medicine, University of Nairobi, Nairobi, Kenya

**Keywords:** Blindness, hypodontia impacted teeth, syndrome

## Abstract

A case is reported of a 26-year-old blind man with hypodontia and multiple apparently underdeveloped impacted teeth. The patient reported that he had progressively developed visual impairment at the age of 11 years whence he became totally blind when he turned 12 years. The aim of this report is to open an academic and professional debate on the challenges of its definitive diagnosis and appropriate intervention.Blindness is not reported in any of the previously described syndromes; therefore, concurrent manifestation of "hypodontia, blindness, failure of eruption and digital lesions" can be proposed as a syndrome. However, in the absence of genetic studies, it is difficult to characterize this case with any one of the specifically documented syndromes; therefore, academic and professional discourse is suggested with regard to appropriate intervention.

## Introduction

Failure of permanent teeth to erupt is one of the commonest dental abnormalities; however, hypodontia arising due to multiple failures of tooth eruption manifesting with total blindness is rare. When there is the clinical absence of one or several teeth and the history indicates that they have not been extracted, then partial anodontia/hypodontia or tooth impaction should be considered.^1^ A generalized failure of tooth eruption due to bony impaction may be associated with diverse syndromic conditions, including amelogenesis and osteogenesis imperfecta, the Gardener’s, Down, Aarsskog, Zimmerman-Laband and Noonan’s syndromes, as well as cleidocranialdysplasia. Blindness is not reported in any of these syndromes; therefore, concurrent manifestation of “hypodontia, blindness, failure of eruption and digital lesions” can be proposed as a syndrome. In order to precisely define and characterize any condition as a syndrome, one should be prudent to perform specific genetic studies that would reveal any aberrant developmental mechanisms within structures such as the jaw bones. The aim of reporting the present case is to initiate some academic and professional discourse regarding what would be its precise diagnosis and appropriate intervention given the available resources.

## Case report

**Figure 1.**
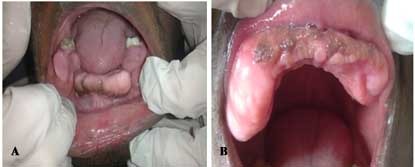
Irregular mandibular arch with eruption of only lower first molars (A). Bulbous maxillary arch and a high-arched palate (B).

**Figure 2.**
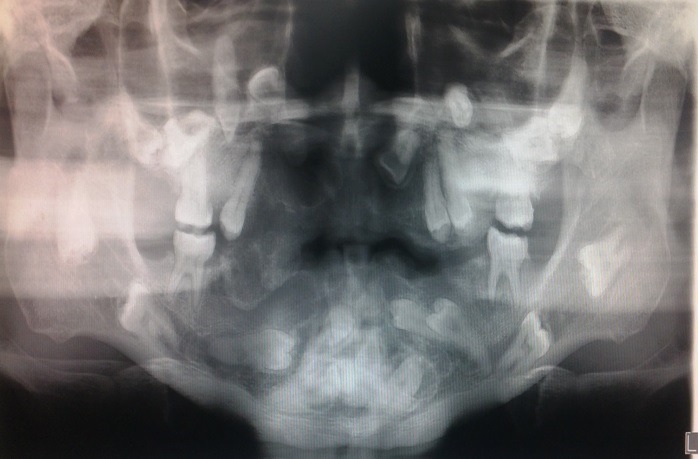
Dental panoramic tomogram showing multiple tooth impactions and irregular bone arches.

**Figure 3.**
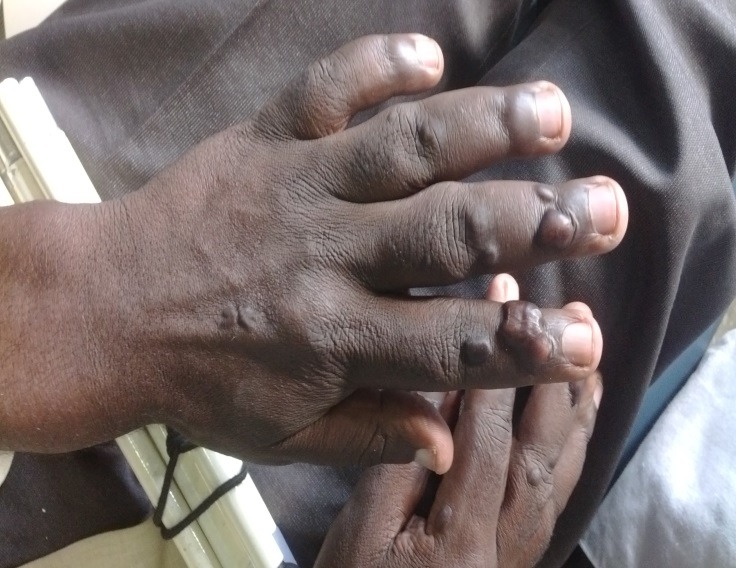
Cutaneous nodular lesions on stumpy fingers.

A 26-year-old blind man presented with a complaint of experiencing functional and aesthetic challenges because, except for his adulthood first molars and one premolar, none of the other teeth had erupted. In addition, the patient reported that he had progressively started losing his eyesight at the age of 11 years whence he became totally blind as he turned 12. On examination, the patient was a well-built young man at a height of 176 cm, weighing 72 kg. He had a broad and flat nasal bridge and thick bushy eyebrows in addition to prominent cheek bones. Ophathamological evaluation revealed whitish discoloration of the ocular mucous membranes of both eyes that were without vision. Oral inspection revealed a high arched palate, remarkably thickened, irregular dental arches with standingfirst permanent molars and a premolar (Figure 1). A dental panoramic tomogram depicted notably malformed dental arches within which the dentition had assumed varied intrabony orientations (Figure 2). General cutaneous inspection elicited pigmented patches of the neck and torso skin. The patient’s fingers were remarkably stumpy with cutaneous nodular lesions (Figure 3) in addition to severely limited mobility of the metacarpo-phalangeal joints. These features were consistent with those of a syndromic condition that would be difficult to clinically characterize and categorize.

## Discussion

Eruption is the occlusal movement of teeth from their developmental position within the jaws to their functional positions in the occlusal plane. This process is guided by the interplay of diverse genetic and physiological mechanisms that are yet to be completely elucidated. Teeth that cease to erupt before emergence in the oral cavity are referred to as impacted.^2^ Current evidence indicates that there are numerous eruption-regulating molecules with similar and overlapping functions which ensure that even the absence of a single factor does not interrupt the eruption process. However, a defect in some of the genes may be responsible for the failure of tooth eruption.^3^Most cases of eruption defects are considered to be part of genetic syndromes; nevertheless, some cases are non-familial, such as primary failure of eruption (PFE) which is marked by failure of permanent teeth to erupt without any associated systemic condition.^4^

The present case exhibited features that should be obviously linked to a syndrome. However, in the absence of genetic studies, it is difficult to associate this case with any one of the specifically documented syndromes. The cause of progressive vision impairment in this case could be logically associated with a non-specific degenerative process of the ocular apparatus responsible for vision. Clinically, this patient manifested features consistent with aberrant developmental genetic mechanisms affecting structures that arise from the ectomesenchyme.

This patient’s desire to access rehabilitative therapy entailing the achievement of a functional and aesthetic dentition would pose significant challenges to any team of clinicians even where all intervention resources may be readily available. The thought of fabricating overdentures would require the creation of a vertical dimension through surgical re-contouring of the arches. Because of the apparently compromised quality of bone within which there are haphazardly impacted teeth, such a surgical procedure may only yield unpredictable results. Academic and professional discourse on cases such as the present one can only be most rewarding among clinicians.

## Acknowledgments

We are most grateful to the administration of the School of Dentistry for the permission to report this case. Our sincere gratitude is extended to the patient for availing all the clinical information. We greatly appreciate the keenness of Miss Josephine W. Dwaluma in preparing this manuscript.

## Authors’ contributions

JM and KS performed the clinical and radiographic examinations, and drafted the manuscript.MC carried out a critical revision of the manuscript. All the authors contributed to final critical revision of the manuscript, and have read and approved the final manuscript.

## Funding

The authors report no funding for this article.

## Competing interests

The authors declare that they have no competing interests with regards to the authorship and/or publication of this paper.

## Ethics approval

The authors declare that the individual whose data is reported in this article has given consent to the authors for the publication of this report.
